# Intergenerational Occurrence of Premature Birth and Reproductive Health in Prematurely-Born Women in the Women’s Health Initiative

**DOI:** 10.1007/s10995-024-03980-w

**Published:** 2024-08-27

**Authors:** Mary C. Sullivan, Pamela L. Brewer, Mary B. Roberts, Robert A. Wild, Aladdin H. Shadyab, Shawnita Sealy-Jefferson, Charles B. Eaton

**Affiliations:** 1 College of Nursing, University of Rhode Island, Providence, RI USA; 2 Care New England Medical Group/Primary Care and Specialty Services, Center for Primary Care and Prevention, Pawtucket, RI USA; 3 Departments of Biostatistics and Epidemiology, Oklahoma City, OK USA; 4 Obstetrics and Gynecology, Oklahoma University Health Sciences Center, Oklahoma City, OK USA; 5 Herbert Wertheim School of Public Health and Human Longevity Science, University of California, San Diego, CA USA; 6 College of Public Health, Ohio State University, Columbus, OH USA; 7 Department of Epidemiology, Brown University, Providence, RI USA; 8 Department of Family Medicine, Warren Alpert Medical School of Brown University, Providence, RI USA

**Keywords:** Preterm birth, Intergenerational, Women’s health, Longitudinal, Women’s Health Initiative

## Abstract

**Objective:**

To compare reproductive history and postmenopausal health by birth status (preterm vs. full term) in a U.S. longitudinal study of postmenopausal women. Birth status was examined according to region of residence, household, and neighborhood socioeconomic status (SES).

**Methods:**

In the Women’s Health Initiative Observational Study, 2271 women were born prematurely (< 37 weeks). ANOVA and Chi-square determined birth status differences of reproductive history, pregnancy, and postmenopausal health. Odds ratios were calculated using either binary logistic or multinomial logistic regression. SES and U.S. region of residence were examined as potential effect modifiers.

**Results:**

Preterm-born women compared to term-born women had higher risk of delivering a premature infant (aOR 1.68, *95% CI* [1.46, 1.93]), higher odds of later-age first pregnancy (aOR 1.27 *95% CI* [1.02, 1.58]), longer duration to become pregnant (> 1 year to pregnancy) (aOR 1.10 *95% CI* [1.01, 1.21]), more miscarriages (aOR 1.23 *95% CI* [1.11, 1.37]), and more pregnancy complications including hypertension (aOR 1.58 *95% CI* (1.13, 2.21)], preeclampsia (aOR 1.64 *95% CI* [1.24, 2.16]), and gestational diabetes (aOR 1.68 *95% CI* [1.11, 2.53]). Preterm-born women had higher odds of menopause before age 50 (aOR 1.09 *95% CI* [1.05, 1.14]). Post-menopause, they had higher rates of diabetes (*p* = .01), hypertension (*p* = .01), hysterectomy (*p* = .045), and higher Charlson Comorbidity Index scores (*p* = .01).

**Conclusions:**

Preterm-born women had higher reproductive and pregnancy risks which when coupled with early menopause, may indicate a shorter childbearing period than term-born women. Guidelines for integration of preterm history in women’s health care across the life course are needed to identify and manage their higher risk.

## Significance

**What is known?:**

Preterm-born individuals have an array of poorer outcomes as adults including cardiovascular, metabolic, renal, respiratory disease, mental health conditions, worse neurodevelopment, and difficulty reaching adult milestones. Yet, little is known about the effect of preterm birth on women’s reproductive health—preconception through menopause and beyond.

**What the study adds:**

This examination of lifelong reproductive health highlights the higher risk for preterm born women calling for the integration of prematurity history in primary and women’s health care.

## Introduction

The stress endured by an infant born before 37 weeks gestation has been recognized among other early life stress factors to have an influence on health across the life course (Halfon et al., 2018). The U.S. rate of preterm birth (PTB; < 37 weeks gestation) increased in 2021 to 10.49% with rates varying by race (Black, 14.75%, Hispanic 10.23%, White, 9.50%, non-Hispanic Asian 9.23%) and more than 95% survive into adulthood (Osterman et al., 2023; Raju et al., 2017). Systematic research around the globe has reported an array of poorer adult outcomes for preterm-born individuals including cardiovascular, metabolic, renal, and respiratory disease, mental health conditions (Crump, 2020), poorer neurodevelopment (Allotey et al., 2018), and difficulty reaching adult milestones (Saigal et al., 2016). In a multinational study of 6.2 million individuals, preterm birth was associated with higher all-cause mortality with higher odds in lower gestations. This risk was higher in premature-born women compared to premature-born men (Risnes et al., 2021).

One area that has received little attention in preterm-adult investigations is women’s reproductive health including pregnancies and pregnancy complications, infant health, and the incidence of reproductive comorbidities. This is an important issue given the economic and social cost of prematurity, the higher risk of pregnancy complications from hypertension and preeclampsia and long-term consequences, and higher risk of offspring morbidity and mortality.

There is evidence of intergenerational occurrence of preterm birth (Swamy et al., 2008; Boivin et al., 2015; Ncube et al., 2017). From Norway’s Medical Birth Registry of 1,167,506 singleton births between 1967 and 1988, Swamy et al.,(2008) found a reduction in the rate of reproduction for those born preterm with an increased risk of preterm birth directly corresponding with lower gestational age categories. In Montreal, Canada, over 7000 prematurely-born women had higher risk for preterm offspring if they were born before 32 weeks gestation [odds ratio (OR) 1.63 for < 32 weeks; OR 1.41 if 32–36 weeks] independent of gestational hypertension and diabetes (Boivin et al., 2015). In a U.S. investigation of 6592 maternal PTB-infant PTB associations, PTB women had increased odds for having a preterm infant (aOR = 1.46, 95% CI 1.0–1.9) with higher risks for earlier gestations versus later gestation (Ncube et al., 2017).

Recent U.S. studies have focused on residential environments revealing social disparities and their contribution to premature birth and generational impacts, though the research is inconsistent. Ncube et al. (2017) reported more preterm birth with non-Hispanic Blacks of both the birth mother and offspring generations, or mothers who moved to neighborhoods with a higher percentage of non-Hispanic Black residents. (Castrillio et al., 2014) using Illinois birth records, found no intergenerational trend for prematurity in non-Hispanic Whites and African American women born small for gestational age. In contrast, Smid et al. (2017) examined preterm birth intergenerational occurrence in 71,676 non-Hispanic White and non-Hispanic Black women in Virginia. After adjustment for confounders, only non-Hispanic Black mothers had increased odds of an early preterm infant. No reports were found on the postmenopausal health of women born prematurely including timing of menopause, problems with breast and/or gynecological health.

Given these gaps in the literature, the first aim of this study was to compare women’s self-reported reproductive history including age of menarche, pregnancy and birth histories including prematurely born offspring, pregnancy complications, and postmenopausal health by birth status (preterm vs. full term). Secondly, we examined the association of birth status with region of birth, region of residence, household and neighborhood socioeconomic status (SES) (Griffin et al., 2013). The sample is from the Observational Study (OS) of the Women’s Health Initiative (WHI), a U.S. longitudinal cohort study of postmenopausal women of race, ethnic and social diversity (Anderson et al 2003; Langer et al 2003). The WHI-OS offers a unique opportunity to answer questions of life-long reproductive health from childbearing to post-menopause of prematurely-born compared to term-born women across the U.S.

## Methods

The WHI longitudinal Observational Study (WHI-OS, *n* = 93,676) was designed to examine a natural history of risk factors for chronic diseases and death in postmenopausal women (Anderson et al., 2003; Langer et al., 2003). Between 1993 and 1998, women from 40 clinical centers across the United States were enrolled. The WHI-OS was the natural control for three clinical trial arms.

### Sample

Postmenopausal women ages 50 to 79 found to be ineligible or unwilling to participate in the clinical trials were invited to be one of 100,000 women enrolled in the OS. Enrollment of a diverse sample of women of racial/ethnic groups was affected. Women were excluded if they had medical conditions of less than 3-year survival, complicating conditions of alcohol, drug dependency or dementia, or planned to move from the area within 3 years.

### Procedure

Standardized, self-report questionnaires included demographic and risk exposure data including reproductive history, family, and medical history. Certified staff obtained physical measurements, including blood pressure, height and weight, and blood samples at the clinic visit. Uniform procedures at all 40 study sites were maintained through a standardized written protocol, centralized training of local clinic staff, local quality assurance activities, and periodic quality assurance visits by the Clinical Coordinating Center (Anderson et al., 2003; Langer et al., 2003)*.* Questionnaires were mailed annually with in-person assessment every three years. All participants provided informed consent using ethics approvals by each center’s Institutional Review Board.

### Measures

The exposure of interest, preterm birth, was determined at study enrollment when participant’s gestational age at birth was queried as full term (pregnancy lasted about 9 months) or preterm (born 4 or more weeks premature). The primary outcomes of interest included age at menarche, pregnancy complications (preeclampsia, gestational diabetes, hypertension during pregnancy, and infant weighing < 5.5 pounds), birth history (age at first birth, age at last birth, number of live births, number of times ever pregnant, duration to become pregnant, miscarriages, stillbirths, child born more than 3 weeks early, and breastfeeding for one month or more) and postmenopausal gynecological health (breast disease, reproductive surgery, depression, depression treatment, menopause before age of 50, hormone therapy). Variables considered as potential model covariates included birth cohort (estimated from 10-year age groups), race, ethnicity, education, income, Body Mass Index (BMI), smoking, Charlson Comorbidity Index (Austin et al., 2015; Charlson et al., 1994), and health insurance. Additionally, region of residence, region of birth, household SES, and neighborhood SES (Griffin et al., 2013) were examined as potential effect modifiers. Adjudication of study outcomes was completed at the local center by an MD who coded test reports, labs, and self-report documents according to WHI definitions (Curb et al., 2003).

### Analysis

**Fig.1:**
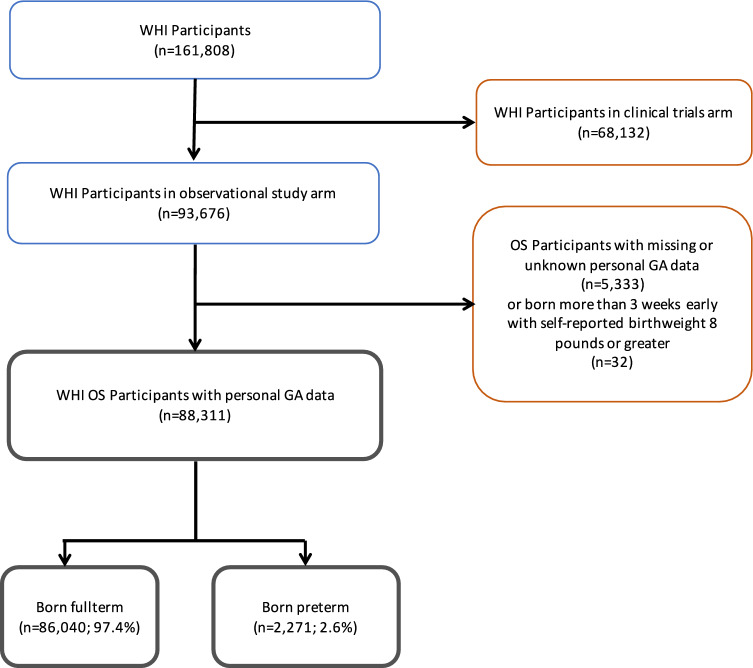
Flow Diagram for WHI-OS Participants

Of 93,676 participants in the WHI-OS, gestational age and birthweights were cross checked leaving 88,311 with reliable gestational age and birthweights. Of these 2.6% (*n* = 2,271) reported being born 4 or more weeks premature (Fig. 1). For the first aim, analyses were performed by birth status (preterm vs. full term). Descriptive statistics by birth status summarized socio-demographic characteristics, reproductive history, pregnancy outcome and postmenopausal gynecological health. Statistical differences by birth status were determined by ANOVA for continuous variables and Chi-square analysis for categorical variables. Odds ratios, both crude and variable-adjusted, were calculated using either binary logistic or multinomial logistic regression depending upon the outcome variable examined. Most outcome variables had two levels and hence binary logistic regression was utilized. However, multinomial logistic regression was used for outcomes of age at menarche, number of times pregnant, number of times pregnant for 6 months or more, age at first birth, age at last birth, length of breastfeeding, and hormone therapy. Covariates of birth cohort (estimated from 10-year age groups), race/ethnicity, education, income, BMI, cigarette smoking, comorbidity index (Charlson Comorbidity Index (Austin et al., 2015; Charlson et al., 1994), and any health insurance were included in full adjusted models.

For the second aim, the potential effect modification of region of residence and SES on birth status, residential region (Northeast, Midwest, West, South), household SES, and neighborhood SES variables were included in the adjusted models as an interaction term with birth status. Household SES was dichotomized into *low* (education less than high school or household income less than $20K/year) and *not low* (high school education or more and household income > $20K/year). Neighborhood SES, an index summary measure developed for the WHI by Griffin et al. (Griffin et al., 2013), was divided into two levels (*above*, *below*) based on median neighborhood SES. U.S. region of residence was based on U.S. Census definition. Statistical differences in region of residence and SES variables were determined by the interaction *p*-value from the multiple variable models. Reproductive health variables by birth status were stratified by SES and region variables. Binary or multinomial logistic regression was used to calculate the odds ratios for birth status within the levels of SES or region variables. Missing data were less than 5%, with most variables having < 1% missingness. Income had the highest amount of missing data (4.3%), therefore the level “missing” was added. Analyses were conducted using SAS v9.4 (Cary, NC). A significance level of *p* ≤ 0.05 was used for all analyses unless otherwise noted.

## Results

We studied women in the WHI-OS who self-reported birth status as full term (*n* = 88,040) or preterm (*n* = 2,271) at baseline assessments (Fig. 1). Preterm-born women were slightly younger (*M* = 62.0, *SD* = 7.3 vs. *M* = 63.5, *SD* = 7.4); *p* = 0.01). The sample was white (term 86%, preterm 87%), Black (term/preterm 8%), Asian (term 3%, preterm 2%), Hispanic (term/preterm 4%) with no differences in race (*p* = 0.16) or ethnicity (*p* = 0.46) by birth status. Preterm-born women had a more college (term 42%, preterm 45%; *p* = 0.01). Household income or household SES, neighborhood SES, U.S. region of birth, U.S. region of current residence or health insurance did not differ by birth status. (Table 1).

**Table 1:** Characteristics of WHI-OS participants by birth status

	Birth Status		
Demographics	Full term (*n* = 86,040)	Preterm (*n* = 2,271)	*P* value
Age (y) (mean, SD)	63.5 (7.4)	62.0 (7.3)	< 0.01
< 50–59	32.05	40.99	< 0.01
60–69	44.03	41.21	
70–79 +	23.92	17.8	
Race (*n*, %)			0.16
American Indian/Alaska	280 (.03)	9 (0.4)	
Asian	2287 (2.7)	45 (2.0)	
Native Hawaiian/Other PI	60 (0.1)	0 (0.0)	
Black	6750 (7.9)	175 (7.6)	
White	73,926 (85.9)	2014 (87.5)	
More than one	873 (1.0)	18 (0.8)	
Unknown/Not reported	1864 (2.2)	42 (1.8)	
Ethnicity (*n*, %)			0.46
Hispanic/Latino	3640 (4.2)	89 (3.9)	
Education level (*n*, %)			< 0.01
< high school graduate	4240 (4.9)	86 (3.7)	
High school graduate	13,787 (16.0)	320 (13.9)	
Some college	31,078 (36.1)	837 (36.3)	
College graduate	36,257 (42.1)	1041 (45.2)	
Missing	678 (0.8)	19 (0.8)	
Income level (*n*, %)			0.65
$50,000 or greater	32,786 (38.1)	901 (39.1)	
$20,000- < $50,000	34,595 (40.2)	921 (40.0)	
< $20,000 per year	12,522 (14.6)	329 (14.3)	
Missing/don’t know	6137 (7.1)	152 (6.6)	
Partnered (*n*, %)	53,417 (62.4)	1477 (64.4)	0.05
Health			
Body Mass Index (mean, SD)	27.5 (6.8)	28.0 (6.7)	< 0.01
Body Mass Index level			< 0.01
Normal (BMI < 25.0)	34,816 (40.5)	845 (36.8)	
Overweight (BMI 25.0–29.9)	28,946 (33.7)	808 (35.2)	
Mild Obese (BMI 30.0–34.9)	13,392 (15.6)	370 (16.1)	
Obese (BMI 35.0 +)	8750 (14.5)	275 (12.0)	
Systolic blood pressure (mm Hg) (mean, SD)	127 (18.0)	127 (17.8)	0.49
Diastolic blood pressure (mm Hg) (mean, SD)	75 (9.3)	75 (9.3)	0.11
Physical activity level (MET-hours/week) (mean, SD)	13.4 (14.5)	13.4 (15.3)	0.80
Smoking status (*n*, %)			0.11
Never smoked	43,055 (50.7)	1181 (52.1)	
Past smoker	36,537 (43.0)	928 (41.0)	
Current smoker	5310 (6.3)	156 (6.9)	
Current smoker	5310 (6.3)	156 (6.9)	
Alcohol servings/week (mean, SD)	2.52 (5.19)	2.58 (5.31)	0.59
Hypertension (*n*, %) +	28,472 (33.1)	851 (37.0)	< 0.01
Diabetes (*n*, %)	3467 (4.0)	133 (5.8)	< 0.01
Cardiovascular disease (*n*, %)	5387 (6.3)	162 (7.0)	0.13
Hyperlipidemia (*n*, %)	12,947 (15.1)	356 (15.5)	0.59
Charlson Comorbidity Index (mean, SD)	29,551 (34.4)	919 (39.9)	< 0.01
Any health insurance (*n*, %)	82,255 (96.6)	2193 (96.3)	0.45

+ systolic blood pressure ≥ 140 mm Hg and/or diastolic blood pressure ≥ 90 mm Hg or being on medication for high blood pressure

### Reproductive Health

**Fig. 2:**
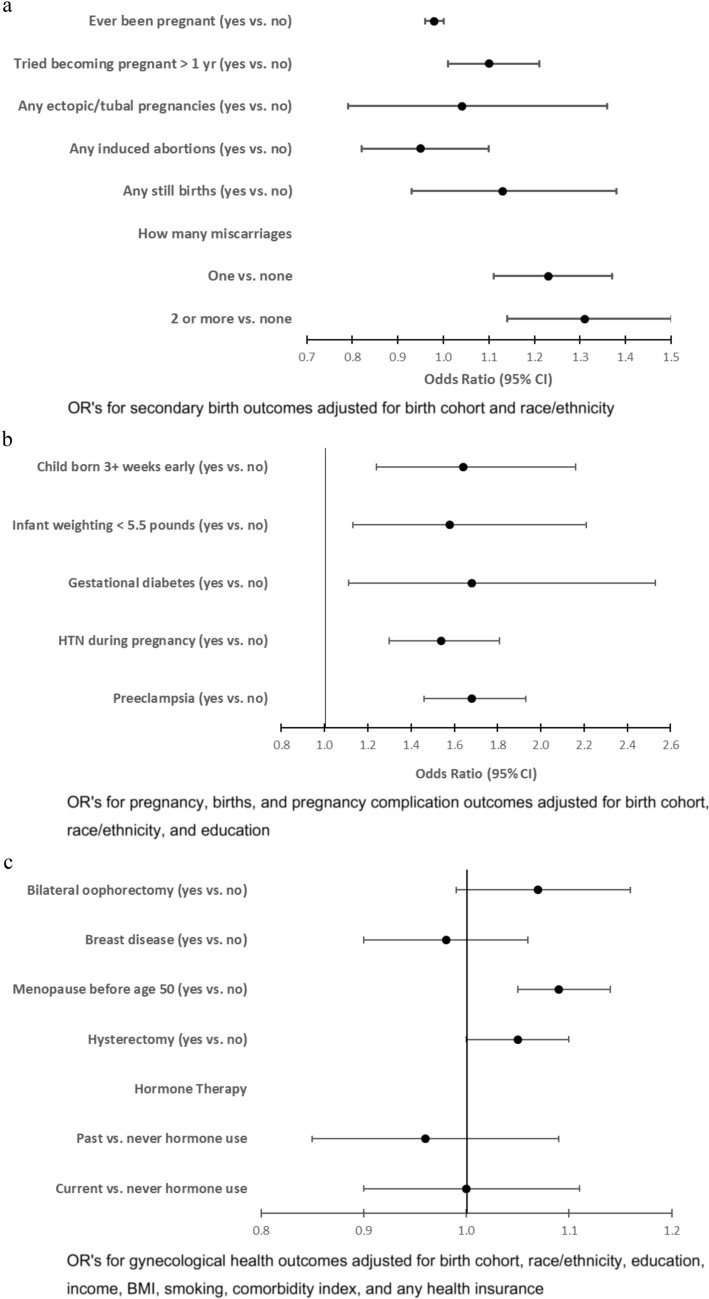
**a** Pregnancy history—odds ratios (95% CI) for preterm vs. full term. **b** Pregnancy complications—odds ratios (95% CI) for preterm vs. full term. **c** Gynecological health—odds ratios (95% CI) for preterm vs. full term

Preterm-born women had 9.9 times the odds of weighing less than 6 lbs. at birth compared to full-term born women [*95% CI* (6.62, 10.19)], 8.46 the odds of being twins or multiple births [*95% CI* (7.44, 9.61)], and 0.72 [*95% CI* (0.71, 0.77)] the odds of having been breastfed. They had non-significant lower odds of ever being pregnant [aOR 0.98, *95% CI* (0.97, 1.00)], took longer than 1 year to become pregnant [aOR 1.10, *95% CI* (1.01, 1.21)], and had fewer pregnancies [OR 1.08*, 95% CI* (0.90, 1.29)] despite no difference in age of menarche. Women preterm-born had higher odds for pregnancy risks than their term-born peers with 1.23 times the odds for one miscarriage [aOR, 1.23, *95% CI* (1.11, 1.37)] and 1.31 times the odds for 2 or more miscarriages [aOR 1.31, *95% CI* (1.14, 1.50)]. In a subsample of women unable to become pregnant (*n* = 427), the preterm-born group had a trend (NS) of increased odds of not being able to conceive [aOR 1.09, *95% CI* (1.00, 1.19)] and increased odds of hormone and/or ovulation problems [aOR 1.53, *95% CI* (1.18, 1.98)]. During pregnancy, the preterm-born women had higher risk for preeclampsia [aOR 1.64, *95% CI* (1.24, 2.16)], hypertension during pregnancy [aOR 1.58, *95% CI* (1.13, 2.21)], and gestational diabetes [aOR 1.68, *95% CI* (1.11, 2.53)]. Their newborn infants were more likely to be premature [aOR 1.68, *95% CI* (1.46, 1.93)] and more likely to weigh < 5.5 lbs. [aOR 1.54, *95% CI* (1.30, 1.81)]. Preterm-born women were less likely to breastfeed their offspring. (Table 2; Figs. 2a, b).

**Table 2:** Reproductive history & gynecological health by birth status

Constructs & variables	Full term population (n = 86,040)	Preterm population (n = 2,271)	Odds ratio (95% CI)	*p-value*	Adjusted odds ratio (95% CI)	*p-value*
Secondary variables						
Weight at birth						
6 pounds or more	70,436 (91.2)	285 (12.8)				
< 6 pounds	6786 (8.8)	1934 (87.2)	9.92 (9.65, 10.20)	< .0001	9.90 (9.62, 10.19)	< .0001
Twin or multiple						
No	84,824 (98.7)	2009 (88.5)				
Yes	1157 (1.4)	260 (11.5)	8.52 (7.49, 9.68)	< .0001	8.46 (7.44, 9.61)	< .0001
Breastfed as Infant						
No	17,839 (25.7)	865 (46.9)				
Yes	51,492 (74.3)	978 (53.1)	0.71 (0.68, 0.75)	< .0001	0.74 (0.71, 0.77)	< .0001
Pregnancy						
Age first period						
11 or less	18,858 (22.0)	558 (24.7)				
12	22,412 (26.2)	588 (26.0)	0.89 (0.79, 1.00)	0.045	0.90 (0.80, 1.01)	0.082
13	24,916 (29.1)	614 (27.2)	0.83 (0.74, 0.94)	0.002	0.86 (0.76, 0.96)	0.009
14	11,313 (13.2)	284 (12.6)	0.85 (0.73, 0.98)	0.026	0.90 (0.77, 1.04)	0.134
15 or greater	8197 (9.6)	217 (9.6)	0.90 (0.76, 1.05)	0.170	0.96 (0.81, 1.12)	0.570
Ever been pregnant						
No	8441 (9.8)	267 (11.8)				
Yes	77,335 (90.2)	1993 (88.2)	0.98 (0.96, 0.99)	0.005	0.98 (0.97, 1.00)	0.014
How many times pregnant						
1	6213 (8.1)	163 (8.2)				
2	17,553 (22.8)	496 (25.0)	1.08 (0.90, 1.29)	0.417	1.07 (0.90, 1.28)	0.454
3	19,493 (25.3)	458 (23.0)	0.90 (0.75, 1.07)	0.232	0.91 (0.76, 1.09)	0.312
4	14,439 (18.7)	389 (19.6)	1.03 (0.85, 1.24)	0.779	1.06 (0.88, 1.28)	0.515
5 or more	19,460 (25.2)	482 (24.3)	0.94 (0.79, 1.13)	0.531	1.02 (0.85, 1.22)	0.871
How many times pregnant 6 mos +						
1	7354 (11.0)	195 (11.1)				
2	20,501 (30.5)	591 (33.7)	1.09 (0.92, 1.28)	0.318	1.08 (0.92, 1.28)	0.353
3	18,700 (27.8)	490 (27.9)	0.99 (0.84, 1.17)	0.890	1.01 (0.86, 1.20)	0.871
4	10,974 (16.3)	279 (15.9)	0.96 (0.80, 1.15)	0.656	1.01 (0.84, 1.22)	0.888
5 or more	9629 (14.3)	201 (11.5)	0.79 (0.65, 0.96)	0.019	0.86 (0.70, 1.06)	0.151
How many tubal pregnancies						
None	84,091 (97.7)	2218 (97.7)				
At least 1	1949 (2.3)	53 (2.3)	1.03 (0.79, 1.35)	0.829	1.04 (0.79, 1.36)	0.796
Any induced abortions						
Pregnant, never had an abortion	65,856 (91.1)	1705 (91.0)				
One or more abortions	6404 (8.9)	169 (9.0)	1.02 (0.88, 1.18)	0.815	0.95 (0.82, 1.10)	0.490
Tried becoming pregnant > 1 yr						
No	70,950 (83.3)	1831 (81.4)				
Yes	14,222 (16.7)	418 (18.6)	1.11 (1.02, 1.22)	0.017	1.10 (1.01, 1.21)	0.029
How many still births						
None	82,670 (96.1)	2175 (95.8)				
At least 1	3370 (3.9)	96 (4.2)	1.08 (0.88, 1.32)	0.452	1.13 (0.93, 1.38)	0.221
How many miscarriages						
None	51,792 (67.3)	1244 (62.6)				
1	16,608 (21.6)	485 (24.4)	1.22 (1.09, 1.35)	0.000	1.23 (1.11, 1.37)	0.000
2 or more	8534 (11.1)	258 (13.0)	1.26 (1.10, 1.44)	0.001	1.31 (1.14, 1.50)	0.000
Births						
Age at first birth						
Never had term pregnancy	2305 (3.3)	68 (3.8)	1.13 (0.89, 1.45)	0.320		
< 20	9677 (14.0)	236 (13.0)	0.94 (0.81, 1.08)	0.364	0.85 (0.64, 1.12)	0.241
20–29	50,452 (73.1)	1313 (72.4)			0.92 (0.72, 1.19)	0.530
30 +	6542 (9.5)	196 (10.8)	1.15 (0.99, 1.34)	0.070	1.09 (0.82, 1.44)	0.571
How many live births						
None	2731 (3.5)	82 (4.1)				
1	8055 (10.4)	220 (11.0)	0.91 (0.70, 1.18)	0.471	0.91 (0.70, 1.18)	0.476
2	22,996 (29.8)	653 (32.7)	0.95 (0.75, 1.19)	0.639	0.94 (0.75, 1.19)	0.630
3	20,811 (26.9)	525 (26.3)	0.84 (0.66, 1.06)	0.148	0.86 (0.68, 1.10)	0.225
4	12,203 (15.8)	296 (14.8)	0.81 (0.63, 1.04)	0.092	0.86 (0.67, 1.10)	0.235
5 or more	10,504 (13.6)	219 (11.0)	0.69 (0.54, 0.90)	0.006	0.76 (0.59, 0.99)	0.043
Number of times ever pregnant						
Never pregnant	8441 (9.9)	267 (11.8)	1.21 (0.99, 1.47)	0.063		
1	6213 (7.3)	163 (7.2)			0.83 (0.68, 1.01)	0.065
2–4	51,485 (60.2)	1343 (59.6)	0.99 (0.84, 1.17)	0.946	0.84 (0.73, 0.96)	0.010
5 +	19,460 (22.7)	482 (21.4)	0.94 (0.79, 1.13)	0.531	0.84 (0.72, 0.98)	0.030
Number of full-term pregnancies						
None	2305 (3.0)	68 (3.4)				
1	7799 (10.1)	207 (10.4)	0.90 (0.68, 1.19)	0.456	0.91 (0.69, 1.21)	0.517
2–4	55,650 (72.3)	1470 (74.0)	0.90 (0.70, 1.15)	0.380	0.93 (0.73, 1.20)	0.582
5 +	11,249 (14.6)	242 (12.2)	0.73 (0.56, 0.96)	0.023	0.82 (0.62, 1.08)	0.149
Age at first term pregnancy						
Less than 20	9729 (14.5)	237 (13.5)				
20–24	31,739 (47.3)	834 (47.6)	1.08 (0.93, 1.25)	0.309	1.09 (0.94, 1.27)	0.269
25–29	19,019 (28.4)	485 (27.7)	1.05 (0.90, 1.23)	0.568	1.08 (0.91, 1.28)	0.369
30–34	5013 (7.5)	149 (8.5)	1.22 (0.99, 1.50)	0.061	1.27 (1.02, 1.58)	0.030
35–39	1583 (2.4)	49 (2.8)	1.27 (0.93, 1.74)	0.132	1.31 (0.95, 1.81)	0.098
Age at last term pregnancy						
Less than 20	898 (1.4)	19 (1.1)				
20–24	7694 (11.5)	220 (12.6)	1.35 (0.84, 2.17)	0.213	1.30 (0.81, 2.10)	0.279
25–29	21,950 (32.9)	562 (32.1)	1.21 (0.76, 1.92)	0.419	1.19 (0.75, 1.89)	0.467
30–34	21,907 (32.9)	580 (33.2)	1.25 (0.79, 1.99)	0.342	1.30 (0.81, 2.07)	0.273
35–39	11,195 (16.8)	291 (16.6)	1.23 (0.77, 1.96)	0.390	1.36 (0.85, 2.19)	0.200
40–44	3000 (4.5)	77 (4.4)	1.21 (0.73, 2.02)	0.456	1.45 (0.87, 2.41)	0.160
Child born 3 weeks' early						
No	22,146 (85.6)	510 (75.9)				
Yes	3714 (14.4)	162 (24.1)	1.68 (1.46, 1.93)	< .0001	1.68 (1.46, 1.93)	< .0001
Breastfed at least 1 month						
Never breastfed	41,485 (49.0)	1186 (53.0)				
1–6 months	21,988 (25.9)	530 (23.7)	0.84 (0.76, 0.94)	0.001	0.86 (0.78, 0.96)	0.005
7–12 months	9504 (11.2)	221 (9.9)	0.81 (0.70, 0.94)	0.005	0.81 (0.70, 0.94)	0.006
More than 12 months	11,779 (13.9)	302 (13.5)	0.90 (0.79, 1.02)	0.095	0.90 (0.79, 1.02)	0.101
Pregnancy Complications						
Preeclampsia						
No	24,344 (95.6)	614 (92.8)				
Yes	1116 (4.4)	48 (7.3)	1.65 (1.25, 2.19)	0.000	1.64 (1.24, 2.16)	0.001
High blood pressure during pregnancy						
No	23,925 (96.7)	617 (94.8)				
Yes	815 (3.3)	34 (5.2)	1.59 (1.14, 2.21)	0.007	1.58 (1.13, 2.21)	0.007
Diabetes during pregnancy						
No	25,386 (98.0)	651 (96.6)				
Yes	513 (2.0)	23 (3.4)	1.72 (1.14, 2.60)	0.009	1.68 (1.11, 2.53)	0.014
Infant weighting < 5.5 pounds						
No	23,043 (88.1)	557 (81.9)				
Yes	3110 (11.9)	123 (18.1)	1.52 (1.29, 1.79)	< .0001	1.54 (1.30, 1.81)	< .0001
Gynecological health						
Bilateral oophorectomy (n, %)						
No	66,875 (79.4)	1738 (77.7)				
Yes	17,342 (20.6)	498 (22.3)	1.08 (1.00, 1.17)	0.050	1.07 (0.99, 1.16)	0.111
Breast disease (n, %)						
No	61,863 (77.6)	1616 (78.1)				
Yes	17,826 (22.4)	453 (21.9)	0.98 (0.90, 1.06)	0.610	0.98 (0.90, 1.06)	0.615
Menopause before age 50 (n, %)						
No	42,804 (51.9)	1015 (46.3)				
Yes	39,674 (48.1)	1178 (53.7)	1.12 (1.07, 1.16)	< .0001	1.09 (1.05, 1.14)	< .0001
Hysterectomy (n, %)						
No	50,160 (58.4)	1273 (56.1)				
Yes	35,804 (41.7)	996 (43.9)	1.05 (1.01, 1.10)	0.029	1.05 (1.00, 1.10)	0.045
Depression (n, %)						
No	71,265 (84.3)	1853 (83.0)				
Yes	13,286 (15.7)	381 (17.1)	1.09 (0.99, 1.19)	0.084	1.04 (0.94, 1.14)	0.471
Depression treatment (n, %)						
No	79,687 (92.6)	2053 (90.4)				
Yes	6353 (7.4)	218 (9.6)	1.30 (1.14, 1.48)	< .0001	1.17 (1.03, 1.34)	0.017
Hormone Therapy (n, %)						
Never used hormones	25,652 (30.4)	655 (29.5)				
Past hormone user	17,802 (21.1)	454 (20.5)	1.00 (0.89, 1.13)	0.985	0.96 (0.85, 1.09)	0.550
Current hormone user	41,044 (48.6)	1110 (50.0)	1.06 (0.96, 1.17)	0.249	1.00 (0.90, 1.11)	1.000

OR’s for secondary birth outcomes adjusted for birth cohort and race/ethnicity

OR’s for pregnancy, births, and pregnancy complication outcomes adjusted for birth cohort, race/ethnicity, and education

OR’s for gynecological health outcomes adjusted for birth cohort, race/ethnicity, education, income, BMI, smoking, comorbidity index, and any health insurance

### Gynecological Health

There were differences in gynecological health. The preterm group was more likely to experience menopause before age 50 years [aOR 1.09, *95% CI* (1.05, 1.14)] and a trend (*p* = 0.045) for higher odds of hysterectomy [aOR 1.05, *95% CI* (1.00, 1.10)] was noted. They were more likely to seek depression treatment [aOR 1.17, *95% CI* (1.03, 1.34)], though the self-report of depression was comparable between birth groups (*p* = 0.47). No higher risk was found for bilateral oophorectomy (*p* = 0.11), breast disease (*p* = 0.615), or hormone therapy (*p* = 0.55) (Table 2; Fig. 2c).

### Effect Modification Models

**Fig. 3:**
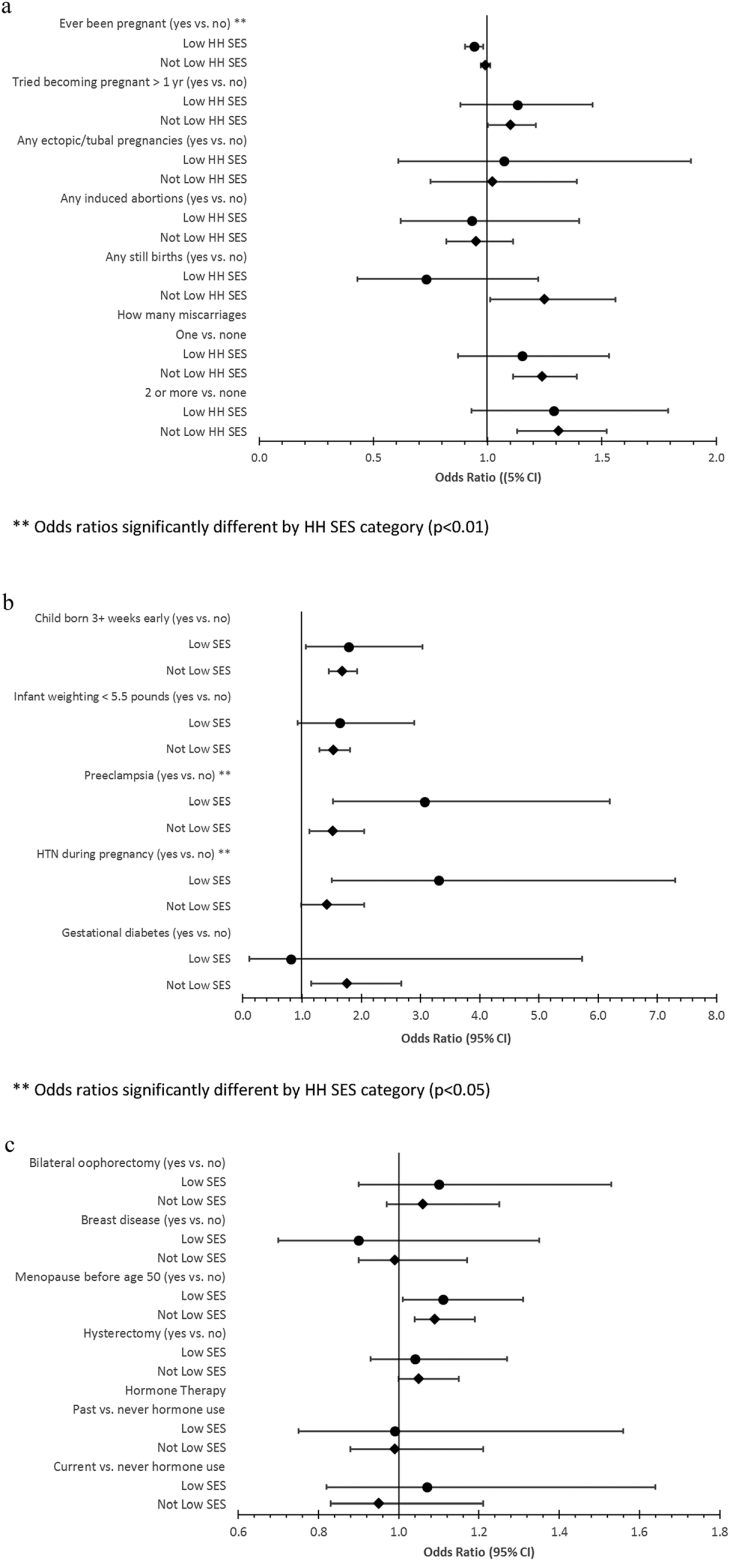
**a** Pregnancy history—odds ratios (95% CI) for preterm vs. full term by household SES status. **b** Pregnancy complications—odds ratios (95% CI) for preterm vs. full term by household SES status. **c** Gynecological health—odds ratios (95% CI) for preterm vs. full term by household SES status

There were significant effect modifications for SES and preterm birth. Women born preterm with low SES had lower odds of ever being pregnant (*p* = 0.0026) compared to their full-term counterparts with low household SES. Preterm women with low SES had greater likelihood of preeclampsia (*p* = 0.0349) and hypertension during pregnancy (*p* = 0.0131) compared to those born full term with low household SES [Table 3; Figs. 3a, b, c]. Effect modification by neighborhood SES had similar results to household SES. Findings did not significantly vary by U.S. region of residence.

**Table 3:** Reproductive history & gynecological health by SES

	Household SES								
	Low household SES				Not low household SES				
Constructs & variables	Full term population (n = 13,814)	Preterm population (n = 347)	Adjusted odds ratio (95% CI)	*p-value*	Full term population (n = 72,226)	Preterm population (n = 1,924)	Adjusted odds ratio (95% CI)	p-value	Interaction p-value
Weight at birth									0.915
6 pounds or more	10,492 (89.0)	38 (11.3)	(reference)		59,944 (91.6)	247 (13.1)	(reference)		
< 6 pounds	1296 (11.0)	299 (88.7)	8.24 (7.70, 8.82)	< .0001	5490 (8.4)	1635 (86.9)	10.29 (9.96, 10.62)	< .0001	
Twin or multiple									0.531
No	13,588 (98.5)	311 (89.6)	(reference)		71,236 (98.7)	1698 (88.4)	(reference)		
Yes	206 (1.5)	36 (10.4)	7.03 (5.01, 9.86)	< .0001	951 (1.3)	224 (11.7)	8.74 (7.60, 10.05)	< .0001	
Breastfed as Infant									0.667
No	2139 (19.2)	126 (45.2)	(reference)		15,700 (27.0)	739 (47.3)	(reference)		
Yes	9023 (80.8)	153 (54.8)	0.70 (0.63, 0.78)	< .0001	42,469 (73.0)	825 (52.8)	0.74 (0.71, 0.78)	< .0001	
Pregnancy									
Age first period									0.381
11 or <	2937 (21.4)	89 (26.0)	(reference)		15,921 (22.1)	469 (24.5)	(reference)		
12	3366 (24.5)	83 (24.2)	0.84 (0.62, 1.15)	0.276	19,046 (26.47)	505 (26.3)	0.91 (0.80, 1.04)	0.168	
13	3636 (26.5)	86 (25.1)	0.82 (0.60, 1.11)	0.191	21,280 (29.6)	528 (27.5)	0.87 (0.76, 0.98)	0.025	
14	2074 (15.1)	39 (11.4)	0.68 (0.46, 1.00)	0.048	9239 (12.8)	245 (12.8)	0.94 (0.81, 1.10)	0.455	
15 or >	1721 (12.5)	46 (13.4)	0.95 (0.66, 1.37)	0.778	6476 (9.0)	171 (8.9)	0.95 (0.79, 1.14)	0.568	
Ever been pregnant									0.003
No	1222 (8.9)	51 (14.9)	(reference)		7219 (10.0)	216 (11.3)	(reference)		
Yes	12,521 (91.1)	292 (85.1)	0.94 (0.90, 0.98)	0.005	64,814 (90.0)	1701 (88.7)	0.99 (0.97, 1.01)	0.180	
How many times pregnant									0.031
1	962 (7.7)	19 (6.5)	(reference)		5251 (8.1)	144 (8.5)	(reference)		
2	2202 (17.6)	53 (18.2)	1.20 (0.70, 2.07)	0.507	15,351 (23.7)	443 (26.1)	1.05 (0.87, 1.27)	0.599	
3	2603 (20.8)	58 (19.9)	1.13 (0.66, 1.94)	0.646	16,890 (26.1)	400 (23.6)	0.88 (0.73, 1.07)	0.209	
4	2260 (18.1)	51 (17.5)	1.16 (0.67, 1.99)	0.599	12,179 (18.8)	338 (19.9)	1.05 (0.86, 1.28)	0.617	
5 or more	4462 (35.7)	110 (37.8)	1.31 (0.79, 2.18)	0.289	14,998 (23.2)	372 (21.9)	0.97 (0.80, 1.18)	0.754	
How many times pregnant 6 mos +									0.042
1	1156 (11.3)	24 (9.6)	(reference)		6198 (10.9)	171 (11.4)	(reference)		
2	2379 (23.3)	59 (23.6)	1.16 (0.71, 1.89)	0.554	18,122 (31.8)	532 (35.3)	1.07 (0.90, 1.28)	0.455	
3	2394 (23.5)	64 (25.6)	1.25 (0.77, 2.03)	0.363	16,306 (28.6)	426 (28.3)	0.99 (0.82, 1.18)	0.876	
4	1855 (18.2)	49 (19.6)	1.21 (0.73, 2.01)	0.453	9119 (16.0)	230 (15.3)	0.98 (0.80, 1.20)	0.869	
5 or more	2413 (23.7)	54 (21.6)	1.14 (0.69, 1.88)	0.601	7216 (12.7)	147 (9.8)	0.81 (0.65, 1.02)	0.072	
How many tubal pregnancies									0.376
None	13,329 (96.5)	335 (96.5)	(reference)		70,762 (98.0)	1883 (97.9)	(reference)		
At least 1	485 (3.5)	12 (3.5)	1.07 (0.61, 1.89)	0.808	1464 (2.0)	41 (2.1)	1.02 (0.75, 1.39)	0.903	
Any induced abortions									0.517
Pregnant, never had an abortion	10,333 (91.7)	247 (91.8)	(reference)		55,523 (91.0)	1458 (90.8)	(reference)		
One or more abortions	942 (8.4)	22 (8.2)	0.93 (0.62, 1.40)	0.726	5462 (9.0)	147 (9.2)	0.95 (0.82, 1.11)	0.534	
Tried becoming pregnant > 1 yr									0.407
No	11,747 (86.5)	283 (84.0)	(reference)		59,203 (82.7)	1548 (81.0)	(reference)		
Yes	1841 (13.6)	54 (16.0)	1.13 (0.88, 1.46)	0.323	12,381 (17.3)	364 (19.0)	1.10 (1.00, 1.21)	0.047	
How many still births									0.236
None	12,941 (93.7)	332 (95.7)	(reference)		69,729 (96.5)	1843 (95.8)	(reference)		
At least 1	873 (6.3)	15 (4.3)	0.73 (0.43, 1.22)	0.228	2497 (3.5)	81 (4.2)	1.25 (1.01, 1.56)	0.040	
How many miscarriages									0.579
None	7912 (63.7)	171 (59.2)	(reference)		43,880 (68.0)	1073 (63.2)	(ref)		
1	2755 (22.2)	70 (24.2)	1.15 (0.87, 1.53)	0.329	13,853 (21.5)	415 (24.4)	1.24 (1.11, 1.39)	0.000	
2 or more	1754 (14.1)	48 (16.6)	1.29 (0.93, 1.79)	0.132	6780 (10.5)	210 (12.4)	1.31 (1.13, 1.52)	0.001	
Births									
Age at first birth									0.705
Never had term pregnancy	395 (3.8)	8 (3.1)	(reference)		1910 (3.3)	60 (3.9)	(ref)		
< 20	2764 (26.3)	56 (22.0)	0.97 (0.46, 2.07)	0.942	6913 (11.8)	180 (11.6)	0.84 (0.62, 1.14)	0.257	
20–29	6540 (62.2)	172 (67.5)	1.29 (0.63, 2.66)	0.490	43,912 (75.1)	1141 (73.2)	0.88 (0.67, 1.15)	0.340	
30 +	819 (7.8)	19 (7.5)	1.18 (0.51, 2.76)	0.697	5723 (9.8)	177 (11.4)	1.06 (0.79, 1.44)	0.684	
How many live births									0.034
None	489 (3.9)	11 (3.8)	(reference)		2242 (3.5)	71 (4.2)	(reference)		
1	1323 (10.6)	27 (9.2)	0.85 (0.41, 1.74)	0.652	6732 (10.4)	193 (11.3)	0.92 (0.69, 1.21)	0.533	
2	2863 (22.8)	68 (23.2)	1.01 (0.53, 1.93)	0.978	20,133 (31.1)	585 (34.4)	0.93 (0.72, 1.20)	0.568	
3	2855 (22.8)	74 (25.3)	1.10 (0.58, 2.09)	0.778	17,956 (27.7)	451 (26.5)	0.83 (0.64, 1.07)	0.155	
4	2203 (17.6)	52 (17.8)	1.02 (0.52, 1.97)	0.965	10,000 (15.4)	244 (14.3)	0.83 (0.64, 1.09)	0.183	
5 or more	2805 (22.4)	61 (20.8)	1.02 (0.53, 1.96)	0.956	7699 (11.9)	158 (9.3)	0.71 (0.54, 0.95)	0.021	
Number of times ever pregnant									< 0.001
Never pregnant	1222 (8.9)	51 (14.9)	(reference)		7219 (10.0)	216 (11.3)	(reference)		
1	962 (7.0)	19 (5.6)	0.49 (0.28, 0.84)	0.010	5251 (7.3)	144 (7.5)	0.91 (0.74, 1.13)	0.408	
2–4	7065 (51.5)	162 (47.4)	0.56 (0.41, 0.78)	0.001	44,420 (61.8)	1181 (61.7)	0.90 (0.78, 10.5)	0.185	
5 +	4462 (32.5)	110 (32.2)	0.64 (0.45, 0.91)	0.013	14,998 (20.9)	372 (19.5)	0.89 (0.75, 1.06)	0.175	
Number of full-term pregnancies									0.027
None	395 (3.2)	8 (2.8)	(reference)		1910 (3.0)	60 (3.5)	(reference)		
1	1264 (10.1)	28 (9.6)	1.07 (0.48, 2.38)	0.878	6535 (10.1)	179 (10.6)	0.89 (0.66, 1.20)	0.438	
2–4	7807 (62.6)	188 (64.6)	1.19 (0.58, 2.44)	0.634	47,843 (74.1)	1282 (75.6)	0.90 (0.69, 1.17)	0.425	
5 +	3001 (24.1)	67 (23.0)	1.19 (0.57, 2.52)	0.643	8248 (12.8)	175 (10.3)	0.76 (0.56, 1.02)	0.068	
Age at first term pregnancy									0.028
< 20	2774 (27.2)	56 (22.6)	(reference)		6955 (12.2)	181 (12.0)	(reference)		
20–24	4602 (45.2)	130 (52.4)	1.39 (1.00, 1.95)	0.054	27,137 (47.7)	704 (46.8)	1.04 (0.87, 1.23)	0.689	
25–29	1983 (19.5)	43 (17.3)	1.11 (0.72, 1.70)	0.636	17,036 (29.9)	442 (29.4)	1.06 (0.88, 1.28)	0.545	
30–34	604 (5.9)	10 (4.0)	0.81 (0.39, 1.68)	0.577	4409 (7.8)	139 (9.2)	1.30 (1.03, 1.64)	0.027	
35–39	227 (2.2)	9 (3.6)	2.19 (1.05, 4.56)	0.036	1356 (2.4)	40 (2.7)	1.19 (0.83, 1.70)	0.350	
Age at last term pregnancy									0.535
< 20	260 (2.6)	6 (2.4)	(reference)		638 (1.1)	13 (0.9)	(ref)		
20–24	1274 (12.6)	37 (14.9)	1.08 (0.45, 2.61)	0.866	6420 (11.4)	183 (12.2)	1.42 (0.80, 2.51)	0.231	
25–29	2886 (28.6)	71 (28.6)	0.93 (0.40, 2.20)	0.875	19,064 (33.7)	491 (32.7)	1.32 (0.75, 2.31)	0.332	
30–34	2952 (29.3)	69 (27.8)	0.91 (0.39, 2.15)	0.829	18,955 (33.5)	511 (34.0)	1.47 (0.84, 2.57)	0.181	
35–39	2040 (20.2)	52 (21.0)	1.08 (0.45, 2.58)	0.861	9155 (16.2)	239 (15.9)	1.51 (0.85, 2.66)	0.158	
40–44	676 (6.7)	13 (5.2)	0.96 (0.36, 2.60)	0.940	2324 (4.1)	64 (4.3)	1.67 (0.91, 3.06)	0.101	
Child born 3 weeks' early									0.944
No	1548 (86.0)	33 (75)	(reference)		20,598 (85.6)	477 (76.0)	(ref)		
Yes	253 (14.1)	11 (25)	1.79 (1.06, 3.03)	0.030	3461 (14.4)	151 (24.0)	1.68 (1.45, 1.93)	< .0001	
Breastfed at least 1 month									0.200
Never breastfed	6259 (46.4)	190 (56.2)	(reference)		35,226 (49.4)	996 (52.4)	(ref)		
1–6 months	3690 (27.3)	70 (20.7)	0.65 (0.49, 0.86)	0.003	18,298 (25.7)	460 (24.2)	0.91 (0.81, 1.02)	0.097	
7–12 months	1561 (11.6)	36 (10.7)	0.80 (0.56, 1.16)	0.237	7943 (11.2)	185 (9.7)	0.82 (0.70, 0.96)	0.015	
> 12 months	1994 (14.8)	42 (12.4)	0.73 (0.52, 1.04)	0.078	9785 (13.7)	260 (13.7)	0.94 (0.82, 1.08)	0.368	
Pregnancy complications									
Preeclampsia									0.035
No	1648 (94.8)	36 (83.7)	(reference)		22,696 (95.7)	578 (93.4)	(reference)		
Yes	91 (5.2)	7 (16.3)	3.07 (1.52, 6.20)	0.002	1025 (4.3)	41 (6.6)	1.52 (1.12, 2.05)	0.007	
High blood pressure during pregnancy									0.013
No	1598 (95.6)	35 (85.4)	(reference)		22,327 (96.8)	582 (95.4)	(reference)		
Yes	74 (4.4)	6 (14.6)	3.31 (1.50, 7.30)	0.003	741 (3.2)	28 (4.6)	1.42 (0.98, 2.05)	0.063	
Diabetes during pregnancy									0.700
No	1760 (97.5)	43 (97.7)	(reference)		23,626 (98.1)	608 (96.5)	(reference)		
Yes	46 (2.6)	1 (2.3)	0.81 (0.11, 5.73)	0.831	467 (1.9)	22 (3.5)	1.76 (1.15, 2.68)	0.009	
Infant weighting < 5.5 pounds									0.780
No	1601 (86.8)	36 (78.3)	(reference)		21,442 (88.2)	521 (82.2)	(reference)		
Yes	243 (13.2)	10 (21.7)	1.64 (0.93, 2.89)	0.087	2867 (11.8)	113 (17.8)	1.53 (1.29, 1.81)	< .0001	
Gynecological health									
Bilateral oopho-rectomy (n, %)									0.689
No	10,389 (78.6)	252 (75.7)	(reference)		56,486 (79.6)	1486 (78.1)	(reference)		
Yes	2826 (21.4)	81 (24.3)	1.10 (0.90, 1.33)	0.364	14,516 (20.4)	417 (21.9)	1.06 (0.97, 1.16)	0.195	
Breast disease (n, %)									0.572
No	10,224 (80.8)	254 (81.7)	(reference)		51,639 (77.0)	1362 (77.5)	(reference)		
Yes	2437 (19.3)	57 (18.3)	0.90 (0.70, 1.15)	0.388	15,389 (23.0)	396 (22.5)	0.99 (0.90, 1.08)	0.810	
Menopause before age 50 (n, %)									0.811
No	5938 (46.6)	133 (39.9)	(reference)		36,866 (52.9)	882 (47.4)	(reference)		
Yes	6801 (53.4)	200 (60.1)	1.11 (1.01, 1.21)	0.029	32,873 (47.1)	978 (52.6)	1.09 (1.04, 1.14)	0.000	
Hysterectomy (n, %)									0.896
No	7287 (52.8)	175 (50.6)	(reference)		42,873 (59.4)	1098 (57.1)	(reference)		
Yes	6519 (47.2)	171 (49.4)	1.04 (0.93, 1.16)	0.476	29,285 (40.6)	825 (42.9)	1.05 (1.00, 1.10)	0.074	
Depression (n, %)									0.445
No	10,033 (75.3)	242 (71.2)	(reference)		61,232 (86.0)	1611 (85.1)	(reference)		
Yes	3291 (24.7)	98 (28.8)	1.06 (0.89, 1.27)	0.518	9995 (14.0)	283 (14.9)	1.03 (0.92, 1.15)	0.657	
Depression treatment (n, %)									0.129
No	12,646 (91.5)	298 (85.9)	(reference)		67,041 (92.8)	1755 (91.2)	(reference)		
Yes	1168 (8.5)	49 (14.1)	1.40 (1.06, 1.84)	0.018	5185 (7.2)	169 (8.8)	1.12 (0.97, 1.30)	0.126	
Hormone Therapy (n, %)									0.768
Never used hormones	5732 (42.3)	129 (37.9)	(reference)		19,920 (28.1)	526 (28.0)	(reference)		
Past hormone user	3485 (25.7)	90 (26.5)	0.99 (0.75, 1.32)	0.950	14,317 (20.2)	364 (19.4)	0.99 (0.88, 1.10)	0.786	
Current hormone user	4328 (32.0)	121 (35.6)	1.07 (0.82, 1.39)	0.621	36,716 (51.8)	989 (52.6)	0.95 (0.83, 1.09)	0.484	

OR’s for secondary birth outcomes adjusted for birth cohort and race/ethnicity

OR’s for pregnancy, births, and pregnancy complication outcomes adjusted for birth cohort, race/ethnicity, and education

OR’s for gynecological health outcomes adjusted for birth cohort, race/ethnicity, education, income, BMI, smoking, comorbidity index, and any health insurance

## Discussion

In this U.S. longitudinal study comparing preterm-born and term-born women’s reproductive health from pre-conception to menopause we found worse pregnancy outcomes, higher likelihood of birthing a preterm infant, earlier menopause and a trend for higher odds of hysterectomy. Preterm-born women had higher risk for a later first pregnancy, a longer time to pregnancy, more miscarriages, and more pregnancy complications of hypertension, preeclampsia (a multisystem disorder including hypertension), and gestational diabetes. These pregnancy risks coupled with early menopause, indicate that preterm-born women have a shorter childbearing period than their term-born peers. For bilateral oophorectomy, breast disease, or menopausal hormone therapy, the preterm and term-born groups were comparable. This study extends prior findings to later ages and shows the significant risks for preterm-born women’s reproductive and gynecological health from menarche to menopause.

The higher rates of pregnancy complications for preterm-born women likely contribute to the risk for delivering a preterm infant. Our findings of higher rates of preeclampsia and gestational diabetes have been reported in younger cohorts. A 1958 British cohort investigation of intergenerational effects on preterm delivery (≤ 36 weeks) identified hypertension history of mother, father and maternal grandmother each independently increased risk of preterm birth (OR 1.7, 2.0, and 1.5 respectively) (Hennessy and Alberman, 1998). This risk was very high for hypertensive preterm-born mothers compared to those born after 36 weeks (21% vs 9%). These pregnancy complications bring higher risk for later morbidity and mortality. In a WHI study of 48,113 women, four pregnancy outcomes (gestational diabetes, hypertensive disorders of pregnancy, low birth weight, preterm delivery) were independently associated with later heart disease (Sondergaard et al., 2020). In our older cohort of postmenopausal women, the health of those preterm-born align with these reports as shown by higher odds for gestational diabetes and hypertension, and higher Charlson Index scores. The utility of the Charlson Index in longitudinal studies is its demonstrated validity to estimate relative risk of death from clinical comorbidities (Austin et al., 2015; Charlson et al., 1994). In a diverse U.S. Collaborative Perinatal Project study of 46,551 women (born 1959–1966), pregnancy complications of preterm delivery, hypertensive disorders of pregnancy, and gestational diabetes were associated with higher mortality 50 years later with higher rates among Black than White participants (Hinkle et al., 2023). Gestational hypertension is associated with a greater risk of later overall cardiovascular disease, coronary heart disease, and heart failure (Lo et al., 2020). Thus, our findings add to the evidence that pregnancy complications experienced by preterm-born women have immediate risk for the health of mother and infant, but also confers long-term risk for chronic disease and early death.

Our findings of higher risk (68%) for preterm-born women to deliver a preterm offspring are comparable to two later cohorts, suggesting some continuity across decades. A 41–63% higher risk for preterm birth < 32 to 36 weeks gestation was reported in a Canadian cohort (Boivin et al., 2015). In Pennsylvania, 46% increase was reported inclusive of birth weight subgroups (Ncube et al., 2017). In Smid et al.’s Virginia cohort (2017), higher intergenerational risk for preterm birth was found for non-Hispanic Black mothers but not non-Hispanic White mothers (i.e., non-Hispanic White, (aOR 1.28, *95% CI* [0.71, 2.31]), non-Hispanic Black (aOR 3.26, *95% CI* [1.77, 6.02]). In contrast, Dorner et al. (2017) found an association for intergenerational preterm birth of < 30 weeks gestation, but no association for preterm births of 34–36 weeks for non-Latino Whites and African–Americans. We did not find higher risk among racialized groups in contrast to findings by Castillio and Smid, though the WHI cohort may have lower power for racial comparisons.

An explanation for higher reproductive risk unexplored in our study is lower social functioning in preterm-born adults. In an international individual participant data (IPD) meta-analysis of 5 preterm-born adult cohorts, lower friend relationships were found, but ratings on partner and family relationships were comparable (Ni et al., 2021). In a small Ontario cohort (*n* = 100 preterm, 89 = term) at age 40, the preterm-born women (birth weight 501-1000 g; birth years 1977–1982) were less likely to have been pregnant (38.3% vs 61.5%) or have children (OR, 0.44; *95% CI* [0.20,0.96]). No differences were found for age of menarche, fertility, miscarriage rate or preterm birth, though their investigation may have been limited by sample size. Behaviorally, both women and men had less dating, marriage or cohabitation which may signal a possible explanation for these pregnancy findings (Saigal et al., 2016).

Socioeconomic factors have been shown to play a role in pregnancy. We found SES to have an effect on pregnancy and pregnancy complications but not postmenopausal health. In an older cohort of individuals born in 1958, the influence of maternal grandmother and mother’s premature status, along with social class were strong predictors of infant birth weight but not gestational age (Emanuel et al., 1992). One population-based study of the heritability of spontaneous prematurity of 2 million Utah births reported a maternal heritability effect of 15.2% with 60.3% due to individual environments (Wu et al., 2015). Household environments may have common patterns across generations that affect women and gestation.

The background in pregnancy and neonatal medical care practices in the U.S. in the mid-1900s is notable for the WHI sample. Women were between 50–79 years of age, with birth dates in the 1920s, 1930s, and 1940s when they enrolled between 1993 and 1998. Between the 1920s and 1940s, hospital births in large cities grew from ~ 50 to 90%. High infant mortality led to the establishment of newborn nurseries but the care of premature infants received little attention before 1950 (Lussky et al., 2005). By the 1950s, a few pediatricians were beginning to create the specialty of neonatology. Incubators for premature babies, originally developed in Europe and Russia, appeared in U.S. exhibits as early as 1898 and showed improved survival, but the carnival atmosphere surrounding exhibitions and misplaced concerns about infections delayed their acceptance by medical professionals and the public (Barr, 1995; Lussky et al., 2005). Thus, if a WHI-OS preterm-born woman was born in a hospital, she likely did not receive specialty care and may have faced a variety of pulmonary complications, thermal irregularities, and nutritional challenges. It is also likely that as premature infants, the WHI participants survived because they had higher birth weights and fewer illnesses than the preterm infants who survive today. While more infants < 32 weeks gestation survive today, for decades the largest group of preterm infant survivors are those born between 34–36 weeks representing more than 70% of preterm infants (Osterman et al., 2023). Thus, our findings are relevant and associated with today’s preterm-born adults, though examination in more racially diverse samples are needed. Future research priorities should include long-term follow-up of SES and racially diverse cohorts.

Our findings align with reports of higher risk indicators for chronic disease as shown by higher odds for gestational diabetes and hypertension, and higher Charlson Index scores. Recently, significant associations were found between preterm birth and prevalent hypertension, earlier onset hypertension, and hypertensive medication use in the WHI-OS participants of the present study (Brewer et al., 2023). Recent calls urge the integration of preterm history in adult care; however there are currently no guidelines addressing this (D’Agata et al., 2022; Luu et al., 2016). Birth history should be included as part of medical records beyond infancy and early childhood. Healthcare providers and the preterm-born individuals themselves need to be aware of their history and long-term risks to begin pro-active intervention.

### Limitations

Limitations of this study include recall bias and self-report. In the WHI-OS, data quality assurance methods were incorporated at several levels and across study sites (Anderson et al., 2003). As an additional step in this study, we cross-referenced self-reported prematurity status with birth weight to define the preterm group. While birth records are generally preferred, the self-report birth data is valid. Birth weight recall by middle-aged and older women was accurately reported by 74% and birth weight category by 87% in a national study (Wodskou et al., 2010). The study outcomes were self-reported and not all were adjudicated which may result in some misclassification. The strengths of this study include its U.S. regional representation, large sample size, extensive baseline data collection, adjudication procedures, and the ability to account for potential confounding variables in analyses. The preterm group size for the not conceiving sub-analysis was small and cautiously interpreted. The details for early birth and newborn care, including family history, social and genetic predispositions are unknown.

### Conclusions for Practice

The Women's Health Initiative (WHI) is a landmark study which has changed women’s health and medical practice around the world. The WHI helps women and their healthcare providers make more personalized and informed decisions about women’s health. Reproductive and gynecological health are central to women across their lifespan. Given the growing evidence for later adult health risks for prematurely born people, birth history should be included in medical records beyond infancy and early childhood. Healthcare providers and the preterm-born themselves need to be aware of the long-term risks to begin pro-active intervention (D’Agata et al., 2022; Kelly et al., 2021; Luu et al., 2016).

## Data Availability

Information on WHI data can be found at whi.org.
